# Development and application of quality assurance methods for interventions in randomised controlled trials of surgical oncology: the ROMIO study (a comparison of minimally invasive and open oesophagectomy)

**DOI:** 10.1038/s41416-025-03236-6

**Published:** 2025-11-25

**Authors:** The ROMIO writing group, Natalie S. Blencowe, Anni King, Beverly Shirkey, Chris Metcalfe, Daisy M. Gaunt, Rachel Brierley, Alex Boddy, Simon Higgs, Simon Dwerryhouse, Paul Wilkerson, Richard Berrisford, Tim Underwood, James Byrne, David Bowrey, Ali Guner, Adam Peckham-Cooper, Andrew D. Hollowood, Renol Koshy, Martin Wadley, Rhys Jones, Arfon Powell, Tanvir Hossain, Heike Cappel Porter, Newton ACS Wong, William B. Robb, Steven Hornby, C. Paul Barham, Jane M. Blazeby, co-applicants and study group, James Byrne, Ben Howes, Chris Rogers, William Hollingworth, Jackie Elliott, Kerry Avery, Jenny Donovan, Lucy Culliford, Marcus Jepson, Peter Lamb, Ravinder Vohra, James Catton, Rachel Melhado, Kishore Pursnani, Richard Krysztopik, Bilal Alkhaffaf, Christopher Streets, Daniel Titcomb, Lee Humphreys, Tim Wheatley, Grant Sanders, Arun Ariyarathenam, Jamie Kelly, Fergus Noble, Graeme Couper, Richard Skipworth, Chris Deans, Anna Paisley, Sukhir Ubhi, Rob Williams, David Exon, Paul Turner, Vinutha Shetty, Ram Chaparala, Khurshid Akhtar, Simon Parsons, Neil Welch, Naheed Farooq, George Hanna

**Affiliations:** 1 Centre for Surgical Research, Population Health Sciences, Bristol Medical School, University of Bristol, Bristol, UK; 2 NIHR Bristol Biomedical Research Centre, Bristol, UK; 3 Bristol Trials Centre, Population Health Sciences, Bristol Medical School, University of Bristol, Bristol, UK; 4 University Hospitals Leicester, Leicester Royal Infirmary, Leicester, UK; 5 Three Counties Oesophago-Gastric Unit, Gloucestershire Royal Hospital, Gloucester, UK; 6 Division of Surgery, University Hospitals Bristol and Weston NHS Foundation Trust, Bristol, UK; 7 University Hospitals Plymouth NHS Trust, Plymouth, UK; 8 University Hospitals Southampton NHS Foundation Trust, Southampton, UK; 9 Division of Upper GI Surgery, Karadeniz Technical University College of Medicine, Trabzon, Turkey; 10 St James’ University Hospital, Leeds, UK; 11 Royal Victoria Infirmary, Newcastle-upon-Tyne, UK; 12 South Tees NHS Trust, Middlesbrough, UK; 13 Cardiff and Vale University Health Board, Cardiff, UK; 14 Department of Cellular Pathology, North Bristol NHS Trust, Bristol, UK; 15 Minimally Invasive Upper GI Surgery Group, Beaumont Hospital, Dublin, Ireland; 16 Department of Health Economics and Health Policy, University of Bristol, Bristol, UK; 17 Patient and Public Contributor, Bristol, UK; 18 Population Health Sciences, Bristol Medical School, University of Bristol, Bristol, UK; 19 Department of General Surgery, Royal Infirmary of Edinburgh, Edinburgh, UK; 20 Trent Oesophago-gastric Unit, Nottingham University Hospitals NHS Trust, Nottingham, UK; 21 Greater Manchester Oesophagogastric Surgery Unit, Northern Care Alliance, Salford, UK; 22 Department of Upper GI Surgery, Royal Lancashire Hospitals NHS Foundation Trust, Preston, UK; 23 Division of Surgery, Imperial College London, London, UK

**Keywords:** Health services, Surgical oncology

## Abstract

**Introduction:**

Results of RCTs are criticised because the quality assurance (QA) of surgical interventions is not considered. This is particularly true in cancer trials, because higher standards of surgery may confer more favourable outcomes. Although methods for surgical QA exist, it is unclear how to operationalise and report them in the context of pragmatic cancer trials. We describe the development and application of QA processes to an RCT comparing laparoscopically assisted (LAO) and open oesophagectomy (OO) in patients with localised oesophageal cancer.

**Methods:**

Three QA measures were developed in Phase 1 and tested for feasibility in Phase 2: (i) centre/surgeon entry criteria, (ii) agreement of key components of LAO/OO, and (iii)monitoring adherence to intervention protocols using CRFs and intra-operative photographs.

**Results:**

All centres met entry criteria and 30/31 Phase 2 surgeons submitted two videos. Although photos were received for 88.8% of procedures, only 44(14.9%) were complete. Adherence to key intervention components (abdominal/thoracic nodal clearance, hiatal dissection) was consistently reported as better in CRFs than that observed in the intra-operative photographs.

**Conclusion:**

Embedding QA measures into pragmatic surgical cancer RCTs is feasible, and provides important data about the quality of interventions. Methods to streamline data collection and analyses are needed prior to widespread use.

## Introduction

Randomised controlled trials (RCTs) in surgical oncology are notoriously difficult to design and conduct, due to numerous methodological and cultural challenges [1]. A major methodological challenge is the risk of introducing performance bias. Surgeons may be more familiar with (or prefer) one intervention compared with another, and therefore deliver it and/or the associated peri-operative care to a different standard than that of the comparator [2]. This is illustrated by an RCT comparing standard (D1) and radical (D2) gastrectomy, which found 53% of patients in the D1 resection group underwent more radical dissection than specified (i.e. more like a D2 operation), and 84% of operations in the D2 gastrectomy group had less dissection than specified (i.e. more like a D1 operation). This cross-contamination between treatment groups undermines the likelihood of detecting any potential therapeutic advantage of the intervention. A potential way of reducing performance bias within surgical RCTs is to embed quality assurance (QA) processes for interventions within the trial, to provide reassurance about the standards of surgery and facilitate replication of successful interventions in practice. This may be particularly important in RCTs involving cancer surgery, where interventions and comparators are often similar to each other (e.g. extent of lymphadenectomy, mode of access) and therefore distinction between them is crucial to ensuring results reflect the intended comparison.

Three distinct categories have been proposed for undertaking QA in surgical RCTs: (i) trial entry criteria for centres and surgeons, (ii) standardisation of surgical techniques and (iii) monitoring of intervention delivery during the trial [3]. Currently, however, the use of QA in surgical RCTs is rare. A systematic review of 80 surgical RCTs found that 18% used entry criteria for surgeons or centres, 29% attempted to standardise the surgical procedures under evaluation, and 28% undertook some form of monitoring of intervention delivery during the trial [4]. Practical, robust approaches to QA in surgical RCTs are therefore required. We have completed a pragmatic surgical RCT comparing minimally invasive (hybrid) oesophagectomy with open surgery (ROMIO: Randomised Oesophagectomy—Minimally Invasive or Open HTA14/140/78) [5, 6], within which we developed methods for assessing surgical QA [7]. Here, we report QA findings and describe the feasibility of embedding these methods into an RCT.

## Methods

The ROMIO study aimed to compare, in patients with cancer of the oesophagus and/or oesophago-gastric junction, the clinical and cost-effectiveness of laparoscopically assisted oesophagectomy (LAO) and open oesophagectomy (OO) in terms of recovery, health-related quality of life, costs and survival. ROMIO was conducted at eight UK surgical centres. During the initial phase (in two centres) the QA methods were developed, with an overall purpose of establishing whether LAO (a newer procedure) was performed to the same surgical standard as OO (a more established procedure) [8]. The QA comprised of three phases: (i) entry criteria for centres and surgeons, (ii) agreement of the key components of LAO and OO and development of intervention protocols and (iii) design of methods for monitoring adherence to the key components. In the second phase, the QA methods were applied across all centres and the feasibility of collecting, transferring, storing and analysing digital data was assessed.

### Entry criteria for centres and surgeons

Two centres, both experienced in minimally invasive oesophagectomy, participated in Phase 1 [9, 10]. Six further UK oesphago-gastric cancer centres were selected to participate in Phase 2, based on surgeons’ interest in participation (a minimum of two surgeons per centre), annual case volume, and evidence of active contribution to national cancer audits [11].

Individual surgeon entry into Phase 2 of the study involved submission of two unedited videos of the abdominal phase of OO and LAO. Surgeons continuing in the study from Phase 1 were encouraged to submit videos, although this was not mandatory. We aimed for videos to be assessed by two surgeons (from a team of seven assessors) using an amalgamated and modified version of the Hierarchical Task Analysis tool for oesophagectomy (HTA-O) [12] and the Objective Structured Assessment of Technical Skills [13] (OSATS) (Appendix 1). The modified tool comprises six skill domains: respect for tissue, time and motion, instrument handling, flow of operation, use of assistants and technical safety, as well as a checklist of procedural components, which was based around the anatomical structures that should be visible if the component had been fully completed. A third assessor was asked to rate the video if either of the original two assessors gave a rating of ‘one’ for the domain ‘technical safety’.

### Agreement and standardisation of key components, and development of intervention protocols

LAO and OO were deconstructed using an existing typology [14] to identify their constituent components. Details of how each component should be standardised (i.e. mandated, optional, flexible or prohibited) within ROMIO were agreed based on existing literature and consensus amongst the study team, and subsequently operationalised into intervention protocols for LAO and OO.

### Monitoring of intervention adherence

Assessment of intervention adherence was undertaken as follows:

*Case report forms* (CRFs) were developed for details of the surgical interventions and histopathology. For the surgical interventions, CRFs were developed from the intervention protocols and completed by surgeons after every procedure. They captured whether the key surgical components were undertaken as intended, and why deviations from the protocol occurred. Histopathological details, including the number of resected lymph nodes and the length of oesophagus resected (measured macroscopically from the resection specimen after formalin fixation), were collected. Histology slides of 10% of the study cases from each centre were reviewed by the Lead Pathologist for ROMIO.

*Intra-operative photographs* were taken of each key component. Photographs were expected to include all anatomical structures that would be visible if the component had been fully completed according to the protocol: left gastric, hepatic and splenic arteries (coeliac axis lymphadenectomy); pericardium, crura, pleural cavities (anterior hiatal dissection); aorta, intersection of the crura (posterior hiatal dissection); carina, bronchi, pulmonary veins, aorta (thoracic lymphadenectomy); thoracic duct (thoracic duct ligation) and anastomosis. Each photograph was assessed by four of a team of 12 surgeons (six consultants and six senior trainees), assigned at random (using Stata statistical software, version 15.1, StataCorp, College Station, Texas, US).

External photographs of the abdominal incisions (including a ruler or feeding jejunostomy plate to facilitate measurement of the wound length) were used to examine adherence to the randomised allocation and length of incisions (i.e. less than 8 cm for LAO, and only one incision for OO). To avoid unblinding of surgeons assessing the surgical photographs (above), wound photographs were assessed separately by the ROMIO research photographer (AK).

### Data collection, transfer and storage

Videos and photographs for LAO were captured using standard laparoscopic stack systems. For filming and photographing OO, various imaging devices were tested. Centres were provided with a written guidance document for all data transfer processes and for the intra-operative photographs; a visual aid with ‘gold standard’ examples was displayed in the operating theatres at each site (Appendix 2). Data collection and transfer was also discussed at annual face-to-face investigator meetings.

Videos and photos were transferred from the laparoscopic stack to an NHS networked computer via an encrypted USB (Universal Serial Bus) drive. Photos were uploaded to the custom-designed study database (Appendix 3), and videos were uploaded to an electronic secure file transfer system. This electronic data transfer method was securely protected through the use of a Hypertext Transfer Protocol Secure extension, facilitating secure sharing and communication over computer networks. The encrypted links were further protected with the use of a password, minimising any information governance risks. All participating sites nominated a designated individual to access the system.

### Data analysis

Only study participants receiving their randomised surgical allocation were included in QA analyses. No formal hypothesis testing was planned or undertaken because of the developmental and exploratory nature of the study methods.

#### Videos

Each skill domain (*n* = 6) within the modified OSATS tool was assessed using a numerical rating scale, of 1–5, 1 being lowest and 5 highest (Appendix 1), with the expectation that a consultant surgeon would score 3 or above for each domain. The hierarchical task analysis component of the modified tool used the classifications ‘not performed’, ‘performed and incomplete’ and ‘performed and complete’.

#### Case report forms

Data from the CRFs were summarised descriptively and tabulated by intervention group.

#### Digital images

Digital photographs were initially assessed for usability by the research photographer (i.e. whether or not a photo of the required structure was provided, and its visual quality, including whether it was assessable) and duplicates were removed. All remaining usable photos were uploaded to a purpose-built electronic platform to facilitate secure assessment (Appendix 3). Surgeon assessors rated these photos according to whether the procedural component was (i) not performed, (ii) performed and incomplete, or (iii) performed and complete, and results were presented by group.

Sensitivity analyses were undertaken to explore the effect on study conclusions of excluding outlying observations: (i) insufficient data (i.e. at least two raters scored the anatomical structure as ‘unable to assess’ either due to a poor-quality photo or a missing structure), and (ii) poor agreement (i.e. where a score of ‘not performed’ and ‘performed and complete’ were both present).

### Ethical approval

The ROMIO study received approval from the South-West Frenchay Research Ethics Committee (REC, study ref: 184167).

## Results

Surgical procedures were completed as assigned in 89.9% (LAO) and 92.4% (OO). Reasons for pre- and peri-operative changes to the protocol are described elsewhere [6].

### Entry criteria for centres

All centres enroled at least two surgeons (range = 3–6) and in five, the whole team participated. The centres all performed at least 50 oesophago-gastric resections annually and entered data into national audits (National Oesophago-Gastric Cancer Audit and NHS Scotland Upper Gastrointestinal Cancer Clinical Quality Performance Indicators).

### Entry criteria for surgeons

We experienced numerous issues when attempting to capture videos of OO, despite involvement of a medical photographer and attempts with various camera equipment and configurations (Table 1). Although we had some success using a laparoscopic camera to record OO cases, this was also fraught with issues, requiring either a designated person to hold the camera (additional team members were usually not available) or an external table-mounted holder, neither of which, together with the laparoscopic equipment itself, were consistently available. Additionally, the camera was sometimes distracting to the surgeons as it was not possible to ensure it was mounted outside their field of view for the entire procedure. Despite assistance from a designated experienced medical photographer, the trial team considered these issues to be insurmountable within the timeframe of the study and the requirement for filming open surgery was dropped.

**Table 1:** Summary of feasibility testing of imaging modalities for open oesophagectomy

Imaging modality	Benefits	Limitations
**Videos**		
Laparoscopic stack	• Readily available at all participating centres. • Surgeons familiar with imaging equipment • Compliant with all infection control policies, allowing it to be inserted directly into body cavities to record high-resolution and well-exposed footage	• Significant distraction to surgeons if mounted in their field of view • External mounting systems not available at all centres • Not all centres able to secure a stack for ‘open’ surgery cases • Mounting systems required significant financial investment and were bulky and incompatible with some ‘stack’ models.
Head-mounted sports camera	• Commercially available and inexpensive (compared to specialised medical loupes) • Provide high resolution imaging from the surgeons’ point-of-view. • Easy-to-use equipment	• ‘Ultra-wide’ field-of-view caused distortion and was too wide to capture the minute details required • Surgeon discomfort • Limited battery life • Lens modifications would be needed • Unable to expose correctly under all lighting, resulting in the need for post-production editing
Miniature ‘bullet’ camera	• Small and specifically designed to film in confined spaces • Allows for high resolution and magnification recordings • Unobtrusive, even if mounted in surgical field	• Manual focusing proved problematic because it was sheathed in a sterile laparoscopic camera sleeve • Image quality compromised by camera sleeve, which could only be rectified by a specialised case that would have required individualised design and manufacturing at considerable cost and time
Integrated surgical light camera	• In-built camera within the operating light handle • High resolution recording, requiring no external equipment	• Surgeons’ heads and hands often obscured the view and required an extra person in theatre to act as camera operator • Not available across all centres
**Photos**		
Laparoscopic stack*	• Sterile and can be inserted directly into cavities to avoid obstructed views • Surgeons are already familiar with this • No extra equipment or personnel required	• Variation in ‘stacks’ make/models and the individualised settings across sites resulted in some low-resolution images • One centre had problems with the availability of laparoscopic equipment for imaging purposes during OO procedures
Digital camera	• Produces high-resolution, detailed images. • Macro lens allows for close-up imaging of small structures • Ring flash illuminated deep within cavities minimising shadows	• Often resulted in obstructed views (especially during the abdominal phase) • Additional personnel required • Potential contamination risk due to use of eternal equipment/photographer • Potential disruption to surgery

*One site was unable to secure a ‘stack’ for OO imaging, due to competing demands. They therefore undertook imaging with the bronchoscope, which was readily available.

Of the 10 surgeons participating in Phase 1, eight continued into Phase 2, with video submissions received from four (two surgeons submitting two videos and two submitting one video). Of the 31 surgeons joining in Phase 2, 30 submitted two videos. It was agreed that the surgeon who did not submit videos was permitted to undertake LAO under the supervision of the surgical lead in that centre. One other surgeon wanted to participate but only performed OO (and did not submit videos) and one surgeon withdrew from the study following video submission. A total of 64 videos were therefore collected, of which 62 were suitable for analysis (one had been extensively edited before submission and the other was a corrupted file; Tables 2 and 3).

**Table 2:** Modified OSATS video assessments*

Skill domain	Definition of score 3	Scores				
		1	2	3	4	5
Respect for tissue	Careful handling of tissue, but occasionally caused inadvertent damage	1	6	57	29	3
Time and motion	Efficient time/motion, but some unnecessary moves	2	19	47	24	4
Instrument handling	Competent use of instruments, but occasionally appeared stiff or awkward	0	5	46	38	7
Flow of operation	Demonstrated some forward planning with reasonable progression of procedure	1	6	54	30	5
Use of assistants	Appropriate use of assistants most of the time	1	6	60	27	2
Technical safety	Potential harms were narrowly avoided	1	5	19	59	12

*We received 62 videos i.e., 2 per surgeon. 32 videos were reviewed by a single assessor, 27 videos were double assessed, two were triple assessed (one because of a score of ‘1’ for technical safety and one when the original intention was for three reviews per video) a further video was assessed by four assessors instead of 2 due to an administrative error.

**Table 3:** Modified HTA-O video assessments*

Operative component	Task (anatomical structure)	Details of completed tasks (%)			
		Not seen in video	Not performed	Incomplete	Complete
Hiatal dissection	Right crus	0	0	4	92
	Left crus	0	0	12	84
	Aorta	0	9	48	39
	Pericardium	0	12	36	48
	Right lung	0	16	40	40
	Left lung	1	19	48	28
Lymphadenectomy	Common hepatic artery	2	30	19	45
	Coeliac artery	2	24	25	45
	Left gastric artery (stump)	2	5	15	74
	Left gastric vein (stump)	2	7	12	75
	Splenic artery	2	27	29	38

*We received 62 videos i.e., 2 per surgeon. 32 videos were reviewed by a single assessor, 27 videos were double assessed, 2 videos were triple assessed and 1 video was reviewed by 4 assessors.

The majority of videos received OSATS scores of three or more, indicating a sufficient standard of surgery (90.7%, Table 2). One video was rated ‘1’ by one assessor for both ‘respect for tissue’ and ‘technical safety’. The second assessor rated these components ‘2’ and ‘3’ respectively. A third and more experienced assessor reviewed and scored the video ‘4’ and ‘3’ respectively. After the discussion, no further action was taken. In terms of HTA-O ratings, dissection of the right crus (*n* = 92, 95.8%) and left crus (*n* = 84, 87.5%) were most often scored as ‘performed and complete’, whereas visualisation of the left lung (*n* = 28, 29.1%) was most often scored as ‘incomplete’ (Table 3).

### Standardisation of surgical techniques and development of the intervention protocols

Deconstruction of LAO and OO identified seven components (Appendix 2). Components agreed as ‘key’ were: (i) incisions and access, (ii) anterior and (iii) posterior hiatal dissection, (iv) abdominal and (v) thoracic lymphadenectomy. Agreed standards for the key components were summarised in the intervention protocols provided to all participating surgeons (Appendices 4 and 5).

### Monitoring adherence to the intervention protocol

#### Case report forms

Completion of CRFs was found to be feasible, with some procedural details recorded for all included patients. Rates of reported adherence were similar for LAO and OO across all key components (Table 4). The highest adherence was dissection of paraoesophageal nodes (LAO 145, 99.3% and OO 149, 100%), and the lowest was en-bloc thoracic lymphadenectomy (LAO 89, 59.3% and OO 100, 67.1%). Adherence was similar between groups across all aspects of the surgical protocol, except procedures to minimise diaphragmatic herniation, which were more commonly performed in the LAO group (LAO *n* = 45, 31%; OO *n* = 21, 14.9%).

**Table 4:** Details of LAO and OO procedures as recorded in case report forms

Procedural component		OO *n* = 149 (%)	LAO *n* = 145 (%)
Number of abdominal incisions	1	126/149 (84.6)	0/145
	2	18/149 (12.1)	0/145
	3	4/149 (2.7)	0/145
	4	1/149 (0.7)	4/145 (2.8)
	5	0/149	103/145 (71.0)
	6	0/149	27/145 (18.6)
	7	0/149	5/145 (3.4)
	8	0/149	6/145 (4.1)
Coeliac axis lymphadenectomy	Common hepatic artery	121/149 (81.2)	114/145 (78.6)
	Left gastric artery	148/149 (99.3)	145/145 (100.0)
	Splenic artery	126/149 (84.6)	118/145 (81.4)
	Coeliac artery	123/149 (82.6)	104/145 (71.7)
	En-bloc removal	123/149 (82.6)	118/145 (81.4)
Anterior hiatal dissection	Removal of crural fibres	114/149 (76.5)	110/145 (75.9)
	Removal of the pericardial fat pad	146/149 (98.0)	143/145 (98.6)
Thoracic lymphadenectomy	Paraoesophageal nodes	149/149 (100.0)	145/145 (100.0)
	Subcarinal nodes	147/149 (98.7)	144/145 (99.3)
	Left main bronchus	116/149 (77.9)	103/145 (71.0)
	Right main bronchus	119/149 (79.9)	113/145 (77.9)
	En-bloc removal	100/149 (67.1)	86/145 (59.3)
Thoracic duct	Ligation of the thoracic duct	134/149 (89.9)	134/145 (92.4)
Anastomosis	Hand-sewn and stapled	8/149 (5.4)	5/145 (3.4)
	Hand-sewn	102/149 (68.5)	102/145 (70.3)
	Stapled	37/149 (24.8)	38/145 (26.2)
	Other	2/149 (1.3)^a^	0/145 (0.0)
Procedure to minimise diaphragmatic herniation	Omentopexy	10/21 (47.6)	18/45 (40.0)
	Narrowing of the hiatus	8/21 (38.1)	13/45 (28.9)
	Sutures between conduit/crus	2/21 (9.5)	8/45 (17.8)
	Colopexy	3/21 (14.3)	12/45 (26.7)
	Other	0/21	1/45 (2.2)^b^
Number of abdominal drains	0	125/149 (83.9)	115/145 (79.3)
	1	23/149 (15.4)	30/145 (20.7)
	2	1/149 (0.7)	0/145 (0.0)
Number of chest drains	1	29/149 (19.5)	32/145 (22.1)
	2	81/149 (54.4)	69/145 (47.6)
	3	39/149 (26.2)	44/145 (30.3)
Gastric drainage procedure	Pyloroplasty	56/65 (86.2)	25/31 (80.6)
	Pyloromyotomy	4/65 (6.2)	2/31 (6.5)
	Thumb/balloon dilatation	1/65 (1.5) 4/65 (6.2)	0/31 4/31 (12.9)
	Botox injection		
Feeding jejuostomy	Intra-operative	58/149 (38.9)	52/145 (35.9)^c^
	Pre-operative	7/149 (4.7)	4/145 (2.8)
Conduit decompression	Naso-jejunal tube	1/149 (0.7)	0/145
	Nasogastric tube	140/149 (94.0)	136/145 (93.8)

^a^Stapled with suture reinforcement.

^b^Pre-existing from previous surgery.

^c^22/52 were laparoscopic.

Full histopathology reports were available and complete for all study participants. The median lymph node harvest for LAO and OO was 23 (range = 5–65) and 25 (range = 8–50), respectively. The median length of the resected oesophagus was 85 mm in both groups, with ranges of 16–230 mm (LAO) and 5–175 mm (OO), providing evidence that the extent of resection was similar in both groups.

#### Digital images

Photographs were obtained for 261 of the 294 patients undergoing the procedure to which they were randomised (LAO = 127, 87.5%, OO = 134, 89.9%). Of 2888 photographs received, 2271 were considered usable after removal of duplicates, additional views and poor-quality images (78.6%; LAO = 1039, OO = 1232). A complete set of images (i.e. all 13 structures included) was provided for 44 of 261 procedures (LAO 27, 18.6% and OO 17, 11.4%). It was not always possible to assess whether the key components had been completed, either because the expected structures were not visible in the image provided, or image quality was poor (Table 5). Overall, OO photographs were scored ‘unable to assess’ (either due to missing structure or poor image quality) more often than LAO photos, with the exception of views of the thoracic duct. More structures were rated as ‘performed and complete’ for LAO procedures than OO: for example, left gastric artery (LAO = 313, 54%; OO = 254, 42.6%), intersection of the crura (posterior hiatal dissection: LAO = 329, 56.7%, OO = 267 44.8%) and the crura during anterior hiatal dissection (LAO = 324, 55.9%; OO = 240, 40.3%) (Table 6).

**Table 5:** Details of photo submissions and suitability for assessment

	OO *n* = 149 (%)	LAO *n* = 145 (%)	Total *n* = 294 (%)
**Number of expected images**^**a**^	1015	1043	2058
**Actual number of images obtained**	1574	1314	2888
Number of intra-operative views	1177 (74.7)	1030 (78.3)	2207 (76.4)
Number of external wound views	397 (25.2)	284 (21.6)	681 (23.5)
**Number of images deemed suitable for assessment**	1232 (78.2)	1039 (79.0)	2271 (78.6)
**Number of procedures with a complete set of images**	17 (11.4%)	27 (18.6%)	44 (14.9%)
**Number of procedures with no images**	15 (10.6)	18 (12.4)	33 (11.2)
**Number of procedures with some assessable images**	134 (89.9)	127 (87.5)	261 (88.7)
**Reasons for a lack of imaging**			
Equipment malfunction	10 (66.7)	15 (83.3)	25 (75.7)
Equipment unavailable	1 (6.7)	0	1 (3.03)
Did not remember	4 (26.7)	3 (16.7)	7 (21.2)
**Number of images unsuitable for assessment (%)**			
Reasons for this:	342	276	618
Duplicate	148 (43.2)	210 (76.0)	268 (43.4)
Unnecessary	96 (28.0)	85 (30.7)	181 (29.2)
Insufficient focus	37 (10.8)	22 (7.97)	59 (9.54)
Insufficient exposure	8 (2.33)	3 (1.08)	11 (1.77)
Obstructed view	43 (12.5)	33 (11.9)	76 (12.2)
Corrupted file	10 (2.92)	13 (4.71)	23 (3.72)

^a^Total expected images calculated with the assumption that the required structures were photographed in the 7 views listed on the ROMIO image reference guide (7 × 294).

**Table 6:** Details of LAO and OO photo assessments*

Structure	Missing or unable to assess due to no structure or poor quality			Not performed			Performed and incomplete			Performed and complete		
	OO	LAO	Total	OO	LAO	Total	OO	LAO	Total	OO	LAO	Total
**Coeliac axis lymphadenectomy**												
Left gastric artery	216 (36.2)	159 (27.4)	375 (31.9)	22 (3.7)	23 (4.0)	45 (3.8)	104 (17.4)	85 (14.7)	189 (16.1)	254 (42.6)	313 (54.0)	567 (48.2)
Hepatic artery	251 (42.1)	245 (42.2)	496 (42.2)	63 (10.6)	104 (17.9)	167 (14.2)	90 (15.1)	94 (16.2)	184 (15.6)	192 (32.2)	137 (23.6)	329 (28.0)
Splenic artery	276 (46.3)	267 (46.0)	543 (46.2)	107 (18.0)	129 (22.2)	236 (20.1)	77 (12.9)	76 (13.1)	153 (13.0)	136 (22.8)	108 (18.6)	244 (20.7)
**Anterior hiatal dissection**												
Pericardium	365 (61.2)	245 (42.2)	610 (51.9)	26 (4.4)	22 (3.8)	48 (4.1)	51 (8.6)	120 (20.7)	171 (14.5)	154 (25.8)	193 (33.3)	347 (29.5)
Crura	272 (45.6)	177 (30.5)	449 (38.2)	13 (2.2)	2 (0.3)	15 (1.3)	71 (11.9)	77 (13.3)	148 (12.6)	240 (40.3)	324 (55.9)	564 (48.0)
Pleural cavities	448 (75.2)	384 (66.2)	832 (70.7)	51 (8.6)	54 (9.3)	105 (8.9)	18 (3.0)	38 (6.6)	56 (4.8)	79 (13.3)	104 (17.9)	183 (15.6)
**Posterior hiatal dissection**												
Aorta (posterior hiatal)	348 (58.4)	232 (40.0)	580 (49.3)	35 (5.9)	35 (6.0)	70 (6.0)	73 (12.2)	134 (23.1)	207 (17.6)	140 (23.5)	179 (30.9)	319 (27.1)
Intersection of crura	266 (44.6)	174 (30.0)	440 (37.4)	10 (1.7)	4 (0.7)	14 (1.2)	53 (8.9)	73 (12.6)	126 (10.7)	267 (44.8)	329 (56.7)	596 (50.7)
**Thoracic lymphadenectomy**												
Carina	337 (56.5)	301 (51.9)	638 (54.3)	9 (1.5)	3 (0.5)	12 (1.0)	46 (7.7)	50 (8.6)	96 (8.2)	204 (34.2)	226 (39.0)	430 (36.6)
Bronchi	354 (59.4)	321 (55.3)	675 (57.4)	20 (3.4)	8 (1.4)	28 (2.4)	45 (7.6)	63 (10.9)	108 (9.2)	177 (29.7)	188 (32.4)	365 (31.0)
Pulmonary veins	391 (65.6)	359 (61.9)	750 (63.8)	30 (5.0)	22 (3.8)	52 (4.4)	27 (4.5)	49 (8.4)	76 (6.5)	148 (24.8)	150 (25.9)	298 (25.3)
Aorta (thoracic)	295 (49.5)	284 (49.0)	579 (49.2)	15 (2.5)	6 (1.0)	21 (1.8)	55 (9.2)	58 (10.0)	113 (9.6)	231 (38.8)	232 (40.0)	463 (39.4)
**Thoracic duct ligation**												
Thoracic duct	282 (47.3)	311 (53.6)	593 (50.4)	8 (1.3)	1 (0.2)	9 (0.8)	23 (3.9)	10 (1.7)	33 (2.8)	283 (47.5)	258 (44.5)	541 (46.0)

*The denominator used to calculate the percentages is the total number of possible ratings. Each participant’s photos were assessed by four surgeons. There were 149 per protocol OO surgeries, and 145 per protocol LOA surgeries. Therefore, for each individual structure, there were 596 possible readings for the OO group and 580 possible readings for the LAO group.

Of the 145 completed LAOs, external wound images were available for 120 (82.7%), although 11 were unusable. The remaining 109 images were used to confirm that the procedure had been undertaken laparoscopically: none depicted an incision larger than 80 mm. Wound images were missing for all three LAO patients, in which the wound length was recorded as greater than 80 mm in the CRFs. Of the 149 completed OO patients, wound images were available for 133 (89.26%), with seven unusable. Of the 23 patients in whom more than one incision was documented in the CRFs, 14 had an accompanying abdominal wound photo of usable quality, confirming these to be the site of drains and/or feeding jejunostomies.

Both sensitivity analyses resulted in similar findings.

#### Comparison of adherence between CRFs and digital images

Although some 31.9% were missing or inconclusive, the rated photographs demonstrated higher rates of incomplete procedures than those reported by surgeons in CRFs. For example, the left gastric artery lymphadenectomy was reported as ‘complete’ for 99.3% of LAO and OO in CRFs, and 42.6% and 54.0% in photographs, respectively. Similar findings were identified for the hepatic artery, splenic artery, pericardium, crura, carina, and thoracic duct (Tables 4 and 6).

### Establishing the feasibility of digital data collection, transfer, storage and analysis

#### Data collection

All centres used a laparoscopic stack system for LAO videos and photos, with the exception of obtaining views of the anastomosis (its position deep in the chest cavity precluded the accurate depiction of colour and vascularity). The requirement to photograph this key component was therefore removed. Although most centres used the laparoscopic stack system to take photos during OO, sometimes this was not available. Feasibility testing of imaging equipment to video/photograph open surgery is summarised in Table 1.

#### Data transfer and storage

Downloading of video data and secure electronic transfer to the ROMIO study team was possible in all centres using the expected methods as outlined in the study protocol.

#### Video assessments

During the collection and sharing of videos, it was feasible to maintain the anonymity of the operating surgeon and centre. The rating process was lengthy, taking many hours per video. Although 37 videos were double reviewed, this became unfeasible and was therefore, after discussion with the trial management group, not completed thereafter.

#### Digital image assessments

618 (21.4%) of the photos were categorised as unusable due to duplicates (deemed as those taken in rapid succession showing no surgical progress) (LAO *n* = 210, OO *n* = 148), unintended additional views (those of adequate technical quality but depicting views outside of the ‘image reference guide’) (LAO *n* = 85, OO *n* = 96) and poor quality images (e.g. unfocused; LAO *n* = 71, OO *n* = 98).

**Fig. 1:**
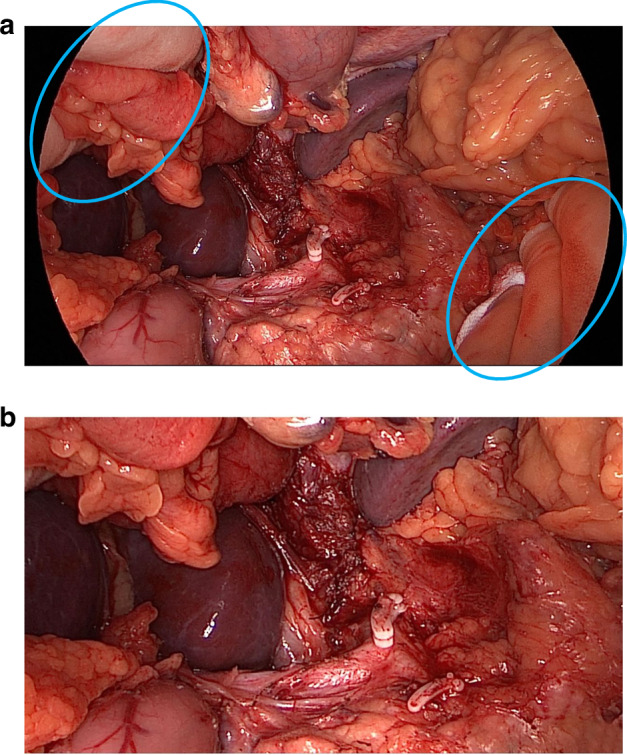
Image anonymisation using ‘cropping’. Image a shows identifiable objects requiring anonymisation, and image b shows the cropped image to achieve this.

**Fig. 2:**
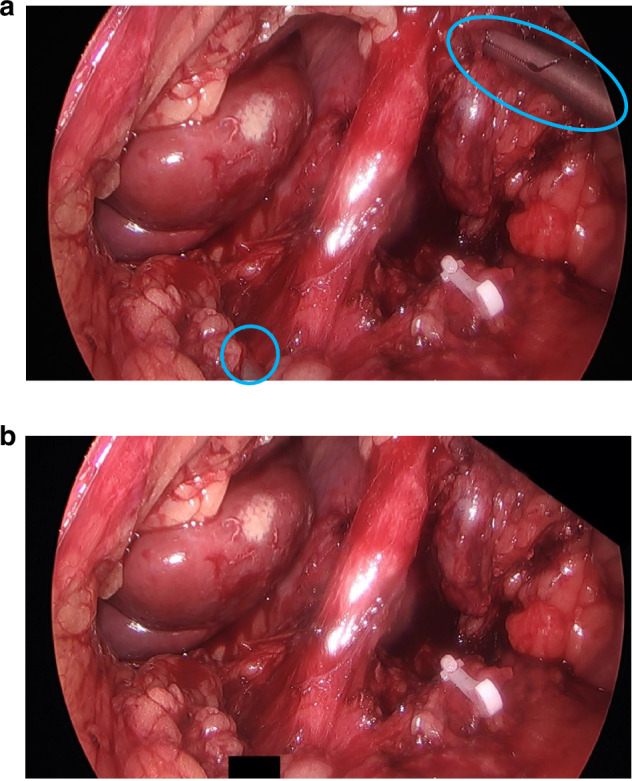
Image anonymisation using ‘black boxes’. Image a shows the identifiable objects requiring anonymisation, and image b shows the black boxes to achieve this.

Another problem was that some photographs revealed the treatment allocation (i.e. whether it was OO or LAO). Common reasons for this were visible procedure-specific surgical instruments or gloved hands (indicating OO). Post-production editing was undertaken to crop identifiers out of view to ensure that assessors could remain blinded (Fig. 1) to the randomised allocation. Occasionally, this was not possible due to the proximity of the identifiers to important anatomical structures. In this scenario, we placed ‘black boxes’ over the identifiers to mask them (Fig. 2). The shape and/or size of the black boxes was purposefully changed throughout to avoid identification of procedure type related to the position of the instruments or hands.

## Discussion

This paper describes the application of QA methods to a surgical RCT, the ROMIO study (laparoscopically-assisted and open surgery for patients with oesophageal cancer [6]). Three QA measures were developed and used: (i) centre and surgeon entry criteria, (ii) development of standardised intervention protocols with mandated, optional, and prohibited components and (iii) methods for monitoring adherence to the protocols. All measures were found to be feasible except for the collection of videos of open surgery, which was abandoned. Although intra-operative photographs were collected for most study participants, the proportion of complete data was low (LAO *n* = 27, 18.6% and OO *n* = 17, 11.4%). For protocol adherence, discrepancies between the CRF and photograph data were consistently observed, indicating the added value of obtaining photographs. Although we recommend that future surgical oncology trials consider using this approach to QA, streamlined methods to improve efficiency are undoubtedly required.

Although the use of QA in surgical RCTs has been recommended and guidance published [12], the challenges in achieving this have been acknowledged [15] and such measures are still infrequently implemented [4]. Recent progress includes the COLOR III trial (comparing transanal total mesorectal excision with laparoscopic approaches for rectal cancer), which included some surgical QA [16]. A Delphi study was performed to prioritise and agree on the standard operative steps, and operative videos collected prior to and during the RCT were used to monitor adherence to the agreed standards. The PANASTA trial (comparing different anastomotic techniques for patients undergoing Whipple procedure) included standardisation of operative steps and submission of intra-operative photos for each randomised patient [17]. The QA confirmed good adherence to the protocol, increasing the validity of the trial findings (no difference between the two techniques). An important consideration when developing QA processes is the need to balance adequate standardisation with the logistics of monitoring adherence, especially within the context of multi-centre RCTs. The QA work in ROMIO aimed to achieve this balance by determining the key components of LAO and OO—and only standardising and monitoring adherence to those key components, as well as providing some flexibility within the standards. This was to optimise the generalisability of results and reflect real-world practice as far as possible, whilst minimising bias. Despite this pragmatic approach, the additional QA processes have the potential to add considerable costs and work to the trial and trial team. In ROMIO, the research photographer was employed full-time throughout the whole trial. We recommend that funding applications for future surgical RCTs include costings for QA processes, including technical expertise and support. Digitalisation and automisation of image capture/assessment may reduce the costs of such QA processes in the future.

Despite its novelty, our study has some limitations. Although development of the QA measures started during the initial phase of the ROMIO study, it was not completed until the main trial was underway. This is partly because the feasibility aspect of the study—overcoming the challenges of collecting and storing high-quality digital data—took longer than expected, and the difficulties in capturing open procedures at multiple sites were insurmountable within the time frame. Although various publications have highlighted that collecting high-quality operative videos of open surgery is possible [18–20], we have not identified any examples where operations occur ‘at depth’ within deep body cavities, similar to the thoracic phase of oesophagectomy. We have recently piloted the use of a remotely controlled compact camera, which can be placed directly above the surgical field and field-of-view adjustments made as required. Although initial testing yielded promising results [21], the camera over-heated during a lengthy procedure, and the need for unscrubbed personnel to control the camera remains an issue. Image assessments may have underestimated the completion of key components, either because structures were obscured by surgeons’ hands or due to poor image quality. A further limitation is that the QA video tool has not been examined for validity or reliability, and raters did not undergo training or inter-rater calibration prior to undertaking assessments. Although the proportion of procedures with accompanying images was high, some were missing, which meant we were unable to ratify the CRF data, including the three LAO patients in whom wound sizes of >8 cm were recorded. Video-imaging of all procedures during the trial may be a preferred method of surgical QA, although this could be more labor-intensive. Although we sought opinions from participating surgeons regarding the standardisation of operative techniques for LAO and OO, we did not attempt to obtain consensus amongst the wider oesophago-gastric community. This may have helped to secure ‘buy in’ in terms of applying the trial results in wider clinical practice and improving the generalisability of findings. Finally, we did not assess whether better scores on video/photo assessments conferred more favourable clinical outcomes, and this warrants future consideration.

Embedding QA processes into surgical RCTs may help to overcome some of the many criticisms levelled at these studies and provide robust data to facilitate the implementation of interventions in practice upon trial completion. It is also possible that surgical QA methods could provide supportive performance measures for centres and surgeons, particularly for those performing significantly poorer than others, although this would need careful validation and monitoring, given that all participating surgeons are fully qualified NHS consultants. It may also be argued that standards for post-operative care pathways need a similar approach. This study has developed and tested processes for the three main types of QA during RCTs: entry criteria for surgeons and centres; standardising techniques and developing protocols; and monitoring intervention delivery. These measures are objective (i.e. observing what surgeons did rather than solely asking them to describe what they did), which provides considerable advantages compared with existing measures, which have historically been largely subjective (e.g. completing CRFs). The main remaining challenges include reducing the logistical burden of QA assessments, improving the quality of digital photos and videos, and improving the availability and archiving of photos and videos for future analysis (both in research and routine practice). Future work is now required to operationalise streamlined QA methods into other surgical contexts.

## Supplementary information

Supplementary Material

## Data Availability

Study data are available on request.
