# Supplementary material for: Development and application of quality assurance methods for interventions in randomised controlled trials of surgical oncology: the ROMIO study (a comparison of minimally invasive and open oesophagectomy)
Source: Br J Cancer. 2025 Nov 25;134(3):404–13. doi: 10.1038/s41416-025-03236-6 (PMC12852800; doi:10.1038/s41416-025-03236-6)
Supplement: Supplementary file 1 — Supplementary Material [file 41416_2025_3236_MOESM1_ESM.docx]

**Supplementary material**

**Appendix 1. Video assessment tool for oesophagectomy**

**a) Please use this tool to rate your overall impression of the video**

| **1** | **2** | **3** | **4** | **5** |
| --- | --- | --- | --- | --- |
| **Respect for tissue** | | | | |
| Frequently used unnecessary force on tissue or caused damage by inappropriate use of instruments |  | Careful handling of tissue but occasionally caused inadvertent damage |  | Consistently handled tissue appropriately with minimal damage |
| **Time and motion** | | | | |
| Many unnecessary movements |  | Efficient time/motion but some unnecessary moves |  | Clear economy of movement and maximum efficiency |
| **Instrument handling** | | | | |
| Repeatedly makes tentative or awkward moves with instruments by inappropriate use of instruments |  | Competent use of instruments but occasionally appeared stiff or awkward |  | Fluid moves with instruments and no awkwardness |
| **Flow of operation** | | | | |
| Frequently stopped operating and seemed unsure of next move |  | Demonstrated some forward planning with reasonable progression of procedure |  | Obviously planned course of operation with effortless flow from one move to the next |
| **Use of assistants** | | | | |
| Consistently placed assistants poorly or failed to use assistants |  | Appropriate use of assistants most of the time |  | Strategically used assistants to the best advantage at all times |
| **Technical safety** | | | | |
| Adverse events that resulted in permanent harm (dangerous) |  | Potential harms were narrowly avoided |  | No adverse events or near misses |

**Did your rating change as the video progressed?**

**Please use this space to document any other comments or concerns**

**b) Please use this tool to rate the ‘completeness’ of each operative phase listed below**

| **Operative phase** | | **Complete** | **Incomplete** | **Not performed** |
| --- | --- | --- | --- | --- |
|  | |  |  |  |
| **Hiatal dissection** | Right crus |  |  |  |
|  | Left crus |  |  |  |
|  | Aorta |  |  |  |
|  | Pericardium |  |  |  |
|  | Right lung |  |  |  |
|  | Left lung |  |  |  |
|  |  |  |  |  |
| **Abdominal lymphadenectomy** | Common hepatic artery |  |  |  |
|  | Coeliac artery |  |  |  |
|  | Left gastric artery (stump) |  |  |  |
|  | Left gastric vein (stump) |  |  |  |
|  | Splenic artery |  |  |  |

**Please use this space to document any other comments or concerns**

**Is there any feedback you’d like to give this surgeon? [Either positive or negative]**

**Appendix 2. Reference guide for the required images of the key components of LAO and OO**


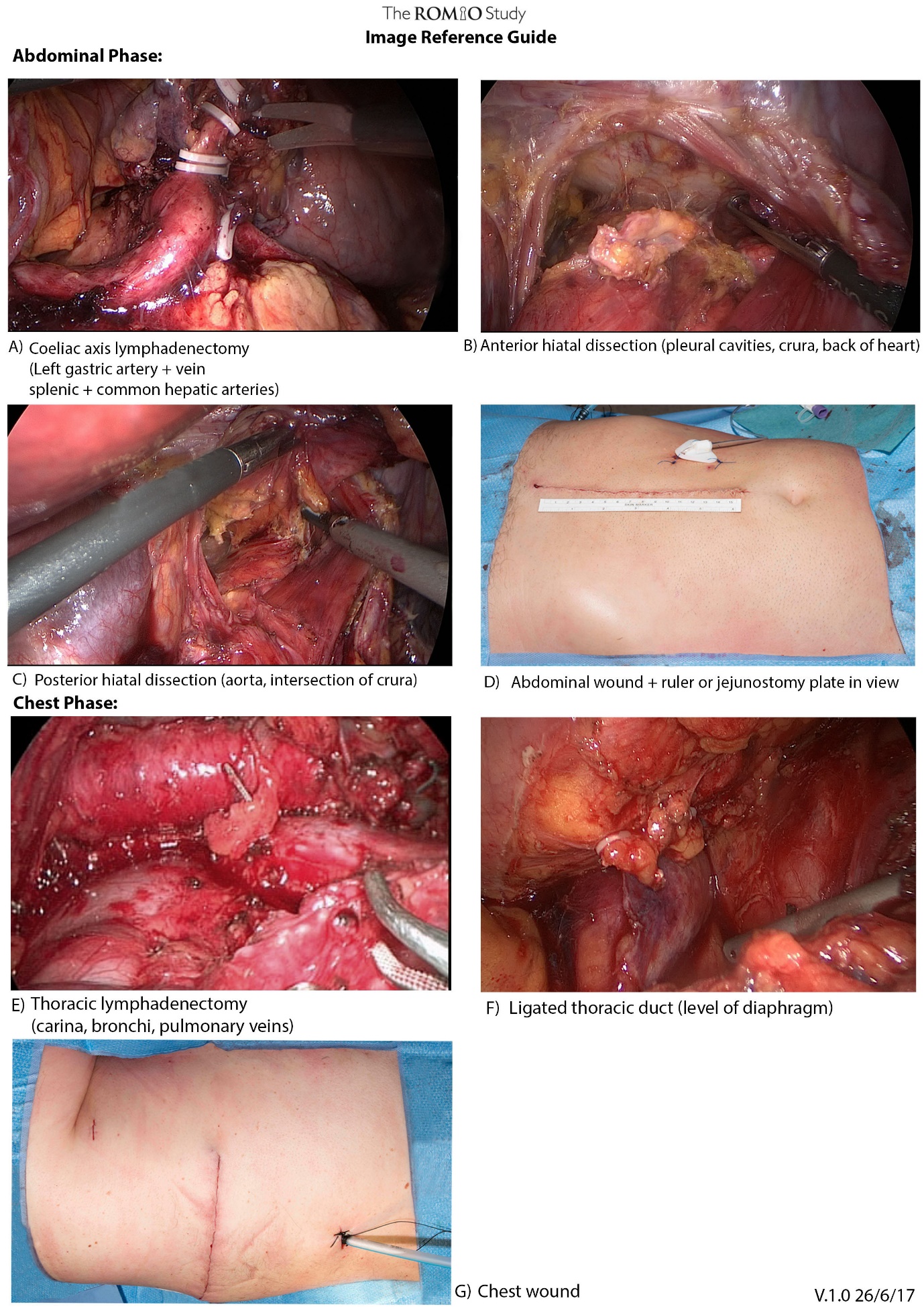


**Appendix 3. Screenshot of the secure electronic platform for image analysis**


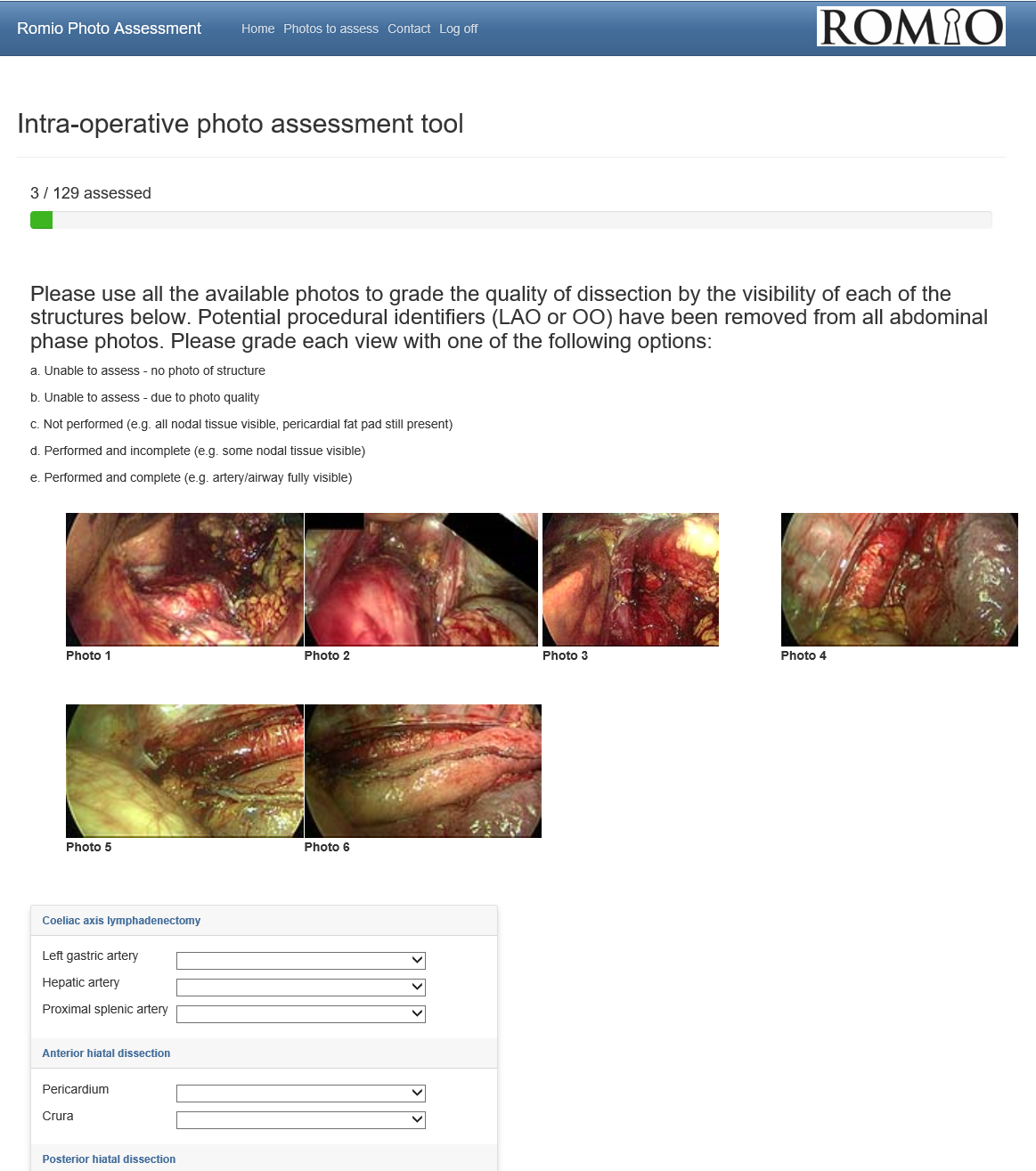


| **Components** | **LAO** | **OO** |
| --- | --- | --- |
| *Incision*  Length of incision(s)  Location of incision(s) | Flexible  [must be <8cm if performing open feeding jejunostomy]  Flexible  [must be port sites only, except for open jejunostomy] | Flexible  Midline or rooftop according to surgeon preference |
| *Access* | Transhiatal and thoracoabdominal approaches prohibited  2 or 3-phase approach allowed, depending on surgeon preference | |
| *Dissection*  Gastric mobilisation  Lymphadenectomies  Removal of:  - pericardial fat pad  - pleura  - crural fibres  - cuff of diaphragm | Based on the right gastroepiploic and right gastric arteries  Along the common hepatic artery, left gastric and splenic artery  either *en bloc* or separately  Mandatory  Mandatory  Mandatory when required for tumour clearance  Mandatory when required for tumour clearance | |
| *Resection*  Transection of the lesser curve | Can be undertaken during abdominal or chest phase, according to surgeon preference | |
| *Reconstruction*  Creation of the gastric conduit  Gastric emptying procedures | Can be undertaken during abdominal or chest phase, according to surgeon preference  Optional. Pyloroplasty/pyloromyotomy both allowed. | |
| *Insertion of surgical adjunct*  Feeding jejunostomy  Naso-jejunal tube  Insertion of drains | Optional (if inserted, can be open or laparoscopic but if open, incision <8cm)  Optional  Optional | Optional  Optional  Optional |
| *Closure*  Abdominal wall/port sites | Technique according to surgeon preference | |

**Appendix 4. Deconstruction of the abdominal phase of open (OO) and laparoscopic (LAO) oesophagectomy into components and establishing standards of surgery for the ROMIO study**

**Appendix 5. Final description of the abdominal phase of open (OO) and laparoscopic (LAO) oesophagectomy, as written in the trial protocol**

1. **Components to be undertaken in the same way for OO and LAO procedures**

Complete gastric mobilisation will be performed based on the right gastroepiploic and right gastric arteries. Pyloroplasty, pyloromyotomy or no drainage is at the surgeon’s discretion. Lymphadenectomies along the common hepatic artery, left gastric and splenic artery either *en bloc* or separately will be performed and removal of sufficient crural fibres and a cuff of diaphragm performed if required for tumour clearance. The pericardial fat pad and strips of pleura will be removed. Transection of the lesser curve may be undertaken or left to the thoracic phase of the operation. Placement of a feeding jejunostomy or naso-jejunal tube is at the surgeon’s discretion as is placement of intra-abdominal and intra-thoracic drains.

1. **Components to be undertaken differently for OO and LAO procedures**

Open oesophagectomy (OO)

The following approaches are permitted: 2-phase (right thoracotomy, laparotomy), 3-phase (right thoracotomy, laparotomy, cervical incision). Use of transhiatal and thoracoabdominal approaches are prohibited. Within these boundaries, the location and length of incisions are at each surgeon’s discretion. Methods to close the incisions are also at the surgeon’s discretion.

Laparoscopically assisted oesophagectomy (LAO)

This operation will consist of identical steps as described above, except that access to the abdominal cavity will be achieved with several 12 or 5mm incisions (as many as needed) and surgery performed laparoscopically. Laparoscopic transhiatal approaches are prohibited. Methods to create the pneumoperitoneum are at the surgeon’s discretion. If a feeding jejunostomy is placed, this may be performed laparoscopically or by creating an additional abdominal incision (maximum length of 8cm).
